# “Beyond the Knife”—Applying Theranostic Technologies to Enhance Outcomes in Neurosurgical Oncology

**DOI:** 10.3390/brainsci14121253

**Published:** 2024-12-13

**Authors:** Santosh Guru, Fred C. Lam, Amirhossein Akhavan-Sigari, Yusuke S. Hori, Deyaaldeen AbuReesh, Armine Tayag, Sara C. Emrich, Louisa Ustrzynski, David J. Park, Steven D. Chang

**Affiliations:** Department of Neurosurgery, Stanford University School of Medicine, Stanford, CA 94305, USA; sg928@cam.ac.uk (S.G.); fredlam@stanford.edu (F.C.L.); asigari@stanford.edu (A.A.-S.); yshori@stanford.edu (Y.S.H.); abureesh@stanford.edu (D.A.); atayag@stanfordhealthcare.org (A.T.); lustrzynski@stanfordhealthcare.org (L.U.); djpark@stanford.edu (D.J.P.)

**Keywords:** brain tumors, gliomas, radiosurgery, Cyberknife, nanotechnology, blood–brain barrier, chemotherapy, neurosurgery, neuro-oncology, image-guided surgery, fluorescence-guided surgery

## Abstract

The current standard of care for brain tumor management includes maximal safe surgical resection followed by concurrent chemotherapy and radiation therapy. Recent advances in image-guided surgical techniques have enhanced the precision of tumor resections, yet there remains a critical need for innovative technologies to further improve patient outcomes. Techniques such as fluorescence image-guided neurosurgery in combination with stereotactic radiosurgery have improved outcomes for patients with brain tumors. In this article for Brain Science’s Special Issue *Recent Advances in Translational Neuro-Oncology*, we review the use of image-guided neurosurgery and stereotactic radiosurgery for the treatment of brain tumors. In addition, we summarize the emerging use of theranostic nanoparticles for the delivery of diagnostic and therapeutic technologies to enable the neurosurgeon to perform more precise surgical resections in the operating room, to specifically target the delivery of existing and novel treatments to tumor cells, and to augment the efficacy of stereotactic radiosurgery. These innovative translational tools will allow neurosurgeons, neuro-oncologists, and radiation oncologists to go “beyond the knife” to improve the survival of brain tumor patients.

## 1. Introduction

**Figure 1:**
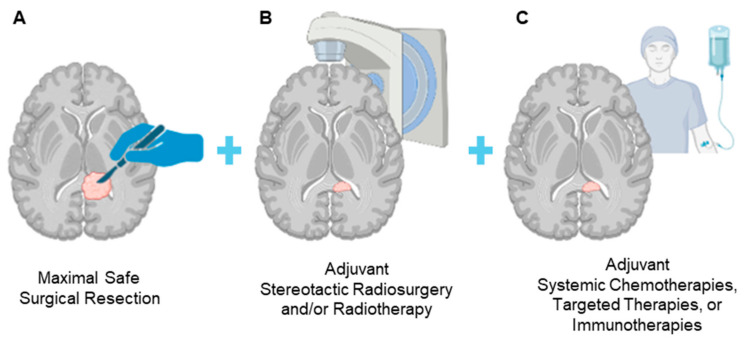
The current standard of care for the treatment of brain tumors. Modern-day treatment of primary and metastatic brain tumors includes: (**A**) maximal safe surgical resection; (**B**) adjuvant stereotactic radiosurgery and/or radiotherapy to the surrounding tumor bed and remaining tumor burden; and (**C**) systemic chemotherapy, targeted therapies, or immunotherapies.

Over the past century, neurosurgical oncology has undergone significant evolution, with maximal safe surgical resection combined with adjuvant therapies now established as the cornerstones of modern treatment (Figure 1). This approach strives to balance the imperative of reducing tumor burden while preserving critical neurovascular functions. Surgical principles for managing central nervous system (CNS) tumors have been explored since antiquity. Ancient Chinese findings from as early as 10,000 BCE describe the management of brain tumors, while trepanned skulls dating back to prehistoric times have been found in France, Mexico, and Peru [1]. The seminal works of William W. Keen, Henry Cushing, and Walter Dandy marked a crucial transition to modern neuro-oncology. William Keen’s pioneering craniotomy in 1888 for successful brain tumor resection set the stage for subsequent advancements in surgical techniques. Cushing further revolutionized neuro-oncology with innovative and meticulous operative methods significantly improving patient outcomes, as documented through his extensive case series of 2000 intracranial tumors [2]. Technological breakthroughs like ventriculography and pneumoencephalography, introduced by Walter Dandy, enabled precise localization of brain tumors through the use of contrast agents and air injected into subarachnoid spaces, thereby enhancing diagnostic accuracy and surgical planning [3,4]. The advent of surgical microscopes by Gazi Yasargil in 1969 further elevated the precision of brain tumor surgery, laying foundational groundwork for the integration of advanced imaging techniques, molecular diagnostics, and targeted therapies in contemporary neurosurgical oncology.

Despite these advancements, significant challenges persist in the standard of care for brain tumor patients. Survival rates for brain tumors remain variable, influenced by tumor-specific factors, patient demographics, and therapeutic variables. For instance, glioblastoma (GBM), the most aggressive primary adult brain tumor, continues to carry a dismal prognosis. Median survival remains approximately 15 months despite aggressive multimodal therapies involving maximal safe resection, adjuvant radiotherapy, and chemotherapy with agents such as temozolomide (TMZ) [5]. The limited success in improving survival outcomes underscores the persistent challenges and limitations inherent in current therapeutic approaches.

One such challenge is the presence of the blood–brain barrier (BBB), which restricts the effective delivery of chemotherapeutics to tumor sites within the CNS [6]. The BBB consists of tight junctions between endothelial cells, astrocyte foot processes, and pericytes. Although small (<400 Da) lipophilic drugs may penetrate the BBB, the delivery of systemic agents such as TMZ remains suboptimal, with cerebrospinal fluid (CSF) concentrations averaging only 20% of plasma levels [7]. The complexity of drug delivery to tumor cells is compounded by the blood–tumor barrier (BTB). This refers to abnormal neovascularization downstream of hypoxia and angiogenic factor release, which can impede chemotherapy entry into tumor areas [8]. Drug efflux transport proteins within the BBB and the rapid clearance of therapeutics via CSF circulation further limit drug efficacy [9]. Novel technologies that can be delivered to tumor cells have the potential to enhance the delivery of therapies across the BBB and BTB and could improve treatment outcomes for brain tumor patients.

## 2. Fluorescence-Guided Brain Tumor Surgery

**Figure 2:**
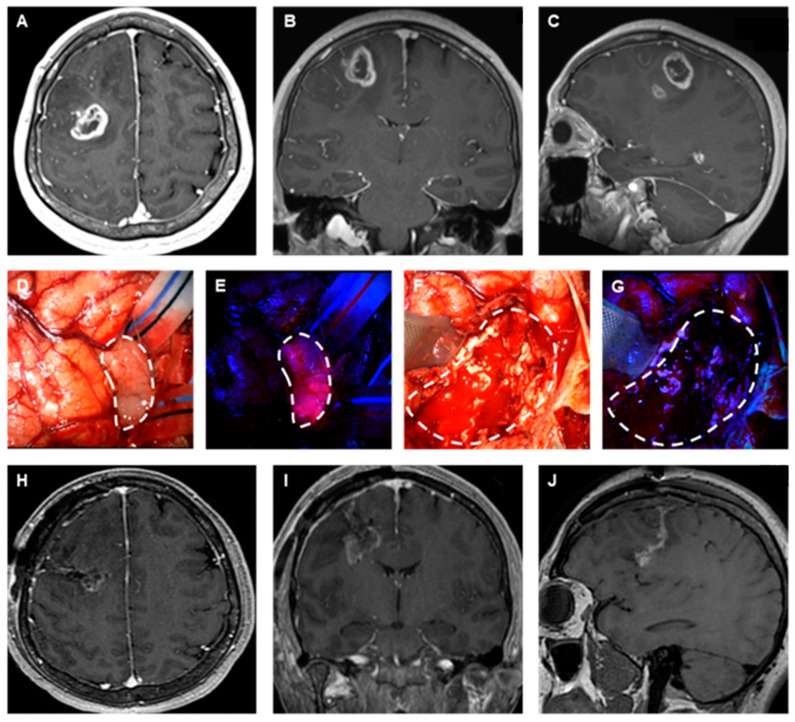
Representative example of fluorescence-guided brain tumor surgery. Preoperative (**A**) axial, (**B**) coronal, and (**C**) sagittal contrast-enhanced T1-weighted magnetic resonance images (MRIs) of a patient with a right frontal glioma. Intraoperative (**D**) white light, and (**E**) 5-ALA fluorescence images of the glioma tumor before resection. Postresection (**F**) white light and (**G**) 5-ALA images of the resection cavity. Postoperative (**H**) axial, (**I**) coronal, and (**J**) sagittal contrast-enhanced T1-weighted MRIs showing maximal safe surgical resection of the glioma. White dashed lines indicated the margins of the tumor.

Our success as tumor surgeons in offering our patients the best outcomes is contingent on our ability to achieve maximal safe resection. However, tumors located in critical or deep brain regions pose significant challenges due to the risk of neurological deficits associated with aggressive resection. Furthermore, the accurate delineation of tumor margins, particularly for diffuse infiltrative tumors such as gliomas, remains a persistent challenge during surgery, often resulting in microscopic residual disease at the tumor periphery [10]. Fluorescence-guided surgery (FGS) has emerged as a promising adjunct to conventional neurosurgical techniques. Fluorescent agents like 5-aminolevulinic acid (5-ALA), which metabolizes into Protoporphyrin IX (PPIX) within tumor cells and fluoresces under blue light, enhance the intraoperative visualization and delineation of tumor tissue and have demonstrated efficacy in increasing the extent of resection in high-grade gliomas [11,12]. High-grade tumors which enhance on MRI following the administration of gadolinium (Figure 2A–C) have a disrupted BBB that also allows for the systemic delivery of 5-ALA to tumor tissue. While the surgeon can differentiate tumor tissue from the surrounding brain under intraoperative white light microscopy (Figure 2D), the use of 5-ALA fluorescence allows the neurosurgeon to clearly visualize the borders of the tumor (Figure 2E). Furthermore, blood in the resection cavity may obscure the ability to see diseased tissue under white light (Figure 2F), but the presence of 5-ALA fluorescence within the resection cavity allows for continued surgical resection (Figure 2G), enabling maximal safe surgical resection (Figure 2H–J).

Indocyanine green (ICG), another fluorescent dye in the near-infrared (NIR) spectrum, exhibits enhanced specificity for tumor cells due to their accelerated endocytosis and disruption of tight junctions, enabling its preferential accumulation within neoplastic tissue [13]. ICG enhances tissue penetration, predicts gadolinium enhancement on postoperative magnetic resonance imaging (MRI), and demonstrates higher sensitivity and negative predictive value compared to 5-ALA in detecting neoplastic tissue [14,15]. The ability to integrate a tumor-targeting diagnostic moiety with a therapeutic agent (a *theranostic*) holds promise in overcoming the conventions of surgical resection followed by adjuvant chemoradiation [16]. However, a recent systematic review of different fluorophores used in fluorescence-guided surgery for gliomas suggests that there is currently not enough evidence supporting the routine use of 5-ALA or sodium fluorescein during surgery because of low fluorescence rates; however, emerging theranostic technologies show promise in overcoming these barriers [17].

## 3. Emerging Uses of Theranostics in Fluorescence-Guided Surgery

Current fluorophores are constrained by limited tissue penetration, specificity, and retention. To address these challenges, novel strategies are emerging, particularly through targeting tumor-specific receptors. This approach has demonstrated the potential to significantly improve tumor specificity. Additionally, the bioconjugation of fluorophores increases their molecular weight, prolonging their half-life [18].

One promising example of this approach is BLZ-100 (Blaze Bioscience, Inc., Seattle, WA, USA), a conjugate of the near-infrared fluorophore ICG with CTX. In a phase 1 trial, BLZ-100 exhibited highly specific localization in both low-grade and high-grade gliomas, with fluorescence retention at the tumor site for over 24 h [19]. This highlights the potential of chlorotoxin-conjugated fluorophores in targeting a broad range of gliomas, independent of tumor grade.

Building on the potential of targeting EGFR, Cetuximab-IRDye800 (UAB Vector Production Facility, University of Alabama, Birmingham, AB, USA)—a fluorescently labeled monoclonal antibody—has demonstrated its ability to provide highly specific contrast in both subcutaneous and orthotopic glioblastoma mouse models [20]. In a first-in-human study, Cetuximab-IRDye800 demonstrated high sensitivity and specificity for tumor tissue under intraoperative near-infrared (NIR) fluorescence imaging, yielding a high tumor-to-background ratio and strong correlation with postoperative histological staining.

Folate receptors, also highly expressed in GBM cells, provide another promising target for immune-conjugated fluorophores [21]. However, their moderate expression in the tumor microenvironment, particularly in tumor-associated macrophages (TAMs), which make up over 50% of the GBM tumor mass, complicates their targeting [22]. To address this, Elechalawar et al., developed carbon nanospheres conjugated with folic acid cationic lipids to target folate receptors [23]. These nanospheres, loaded with the fluorophore 1,1-dioctadecyl-3,3,3,3-tetramethylindotricarbocyanine iodide (DIR), demonstrated effective blood–brain barrier penetration and the ability to target both GBM cells and TAMs. In orthotopic and subcutaneous mouse models, the nanospheres exhibited significantly higher accumulation in tumor tissues compared to non-conjugated nanospheres or the fluorophore alone, underscoring the potential of dual-targeting strategies to maximize tumor resolution.

Integrins, particularly αvβ3 and αvβ5, are also upregulated in GBM, making them another viable target [24]. The arginine–glycine–aspartic acid (RGD) sequence has shown high specificity for targeting these integrins. In a study by Huang et al., the RGD-conjugated fluorophore IRDye 800CW-RGD selectively accumulated in tumor tissue, with prolonged retention, minimal autofluorescence, and precise delineation of tumor margins, as demonstrated by a high tumor-to-background ratio [25].

Similarly, the gastrin-releasing peptide receptor (GRPR) has shown potential as a target for immune-conjugated fluorophores. Li et al., developed 68 Ga-IRDye800CW-BBN, a dual-modality PET/near-infrared fluorescence probe targeting GRPRs [26]. In a first-in-human study involving 14 GBM patients, intraoperative fluorescence correlated strongly with preoperative PET signals and postoperative histopathological analysis, allowing for a clear distinction between tumor tissue and adjacent brain tissue.

**Figure 3:**
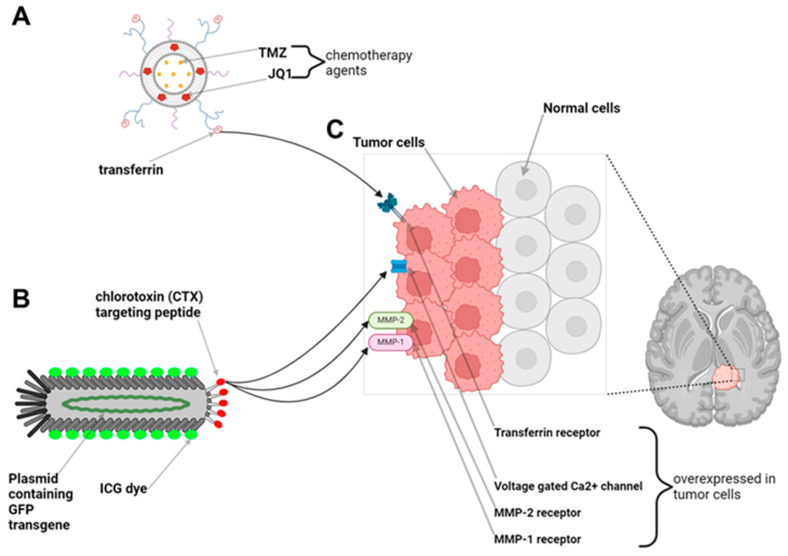
Examples of brain tumor targeting theranostic nanotechnologies. (**A**) Liposomal nanoparticles or (**B**) filamentous phage nanoparticles can be functionalized with surface ligands (i.e., transferrin or chlorotoxin) that recognize (**C**) receptors expressed on the surface of brain tumors (i.e., transferrin receptor, MMP1/2 receptors, and voltage-gated Ca^2+^ channels) for tumor-specific targeting. The conjugation of fluorescent dyes (i.e., ICG) on the surface of the nanoparticles can aid in intraoperative detection of tumor tissue. The packaging of therapies (i.e., chemotherapies, gene therapies) in the nanoparticles allows for concurrent treatment delivery.

We previously published on two different theranostic tools with significant translational potential for use in neuro-oncology. One technology involves the formulation of liposomal nanoparticles which are capable of packaging water-soluble small molecules such as TMZ in their aqueous centers and hydrophobic small molecules such as the bromodomain inhibitor JQ1 in their lipid bilayers, allowing for the delivery of dual combination therapies (Figure 3A) [27]. These liposomal nanoparticles can be further functionalized on their surface with proteins such as transferrin, which have been shown to enable receptor-mediated transcytosis across the BBB, and fluorophores, allowing for fluorescence detection. In an intracranial orthotopic xenograft mouse model of GBM, we demonstrated that these transferrin-functionalized nanoparticles could cross the BBB and BTB, attaching to the surface of intracranial glioma tumors which inherently overexpress transferrin receptors on their cell membranes (Figure 3C, transferrin receptors) [27]. The ability to achieve tumor-specific delivery of combination therapies across the BBB led to decreased tumor burdens, prolonged surgical outcomes, and a relative reduction in systemic drug toxicity profiles in glioma-bearing mice. The second theranostic tool leverages filamentous M13 bacteriophage as a theranostic for tumor imaging in the short-wave infrared (SWIR) spectrum using a patient-derived orthotopic xenograft mouse model of GBM (Figure 3B). Filamentous phage particles are narrow in diameter (5 nm), modular in length, and genetically tunable with the ability to express transgene plasmids. Similarly to liposomal nanoparticles, phage particles have surface peptides that can be conjugated with fluorophores and small molecules. We produced ultrashort (50 nm) M13 “inho” phage particles that expressed the 28-amino acid chlorotoxin (CTX) peptide, known to recognize the MMP1/2 receptors on the surface of glioma cells [28]. We then conjugated ICG fluorophores onto the surface of inho phage particles and delivered them intravenously in a patient-derived xenograft mouse model of GBM, enabling intracranial detection of brain tumors in mice using a SWIR imaging system (Figure 3C) [28]. By integrating receptor-specific targeting with advanced imaging technologies, these strategies offer promising solutions to overcome the limitations of conventional fluorophores, improving tumor visualization during resection and facilitating maximal safe surgical removal. Taken together, our ability to combine existing advanced intraoperative neurosurgical techniques with translational theranostic technologies may allow us to address the large unmet need in offering our brain tumor patients significant survival benefits. Again we provide these findings in tabular form below for our readers (Table 1).

## 4. Emerging Uses of Theranostics in Brain Tumor Imaging

Magnetic resonance imaging (MRI) remains the gold standard modality for brain tumor imaging. This includes functional MRI (fMRI) and diffusion tensor imaging (DTI) modalities which can be used to further characterize the heterogeneous nature of GBM tumors to help differentiate GBM subtypes and has the potential for use in designing personalized therapies based on the unique imaging characteristics of a patient’s tumor [33]. Currently, gadolinium-based contrast agents are routinely used to image the borders of high-grade lesions. However, these agents do not cross the BBB, with signal change dependent upon BBB disruption surrounding tumors. This non-specific accumulation in areas of BBB disruption can lead to false-positive contrast enhancement, blurring the distinction between tumor tissue and inflamed margins [34]. Additionally, gadolinium-based agents have a short half-life, necessitating repeated injections and higher dosages to maintain adequate tumor visualization. To address these challenges, targeted nanoparticle platforms are being developed to extend signal enhancement duration and improve tumor border delineation.

Superparamagnetic iron oxide nanoparticles (SPIOs) have been extensively investigated for their ability to specifically deliver contrast agents. In a study by Sun et al., CTX-targeted iron oxide nanoparticles were tested both in vitro, using the Rat 9L/lacZ glioma (9L) and human D283 medulloblastoma cell lines, and in vivo, using glioma xenograft mouse models [35]. T2-weighted MRI demonstrated a three-fold increase in intensity with CTX-conjugated nanoparticles compared to non-conjugated counterparts. In vivo, the conjugated nanoparticles showed preferential accumulation in tumor sites, accumulating for significantly longer durations than the non-conjugated nanoparticles. This extended accumulation was attributed to enhanced internalization of the conjugated nanoparticles by tumor cells. However, SPIOs are associated with negative contrast effects and magnetic susceptibility artifacts. As negative contrast agents, SPIOs create regions of hypointensity on T2-weighted MRI images, which can be difficult to differentiate from surrounding areas, including hemorrhages, calcifications, and hemosiderin deposits. Moreover, susceptibility artifacts can distort background imaging, complicating interpretation [36].

Targeted nanoparticles that provide positive contrast may overcome these limitations. EGFR, which is overexpressed in 50–60% of glioblastomas and is minimally expressed in normal brain tissue [37], is an attractive target for precise tumor visualization. In a study by Na et al., magnesium oxide (MNO) nanoparticles conjugated with Herceptin, an antibody targeting the Her-2/neu receptor, were used to target EGFR [38]. In a mouse model of brain metastases, these functionalized MNO nanoparticles enabled accurate tumor margin delineation on T1-weighted MRI. Notably, while both functionalized and non-functionalized MNO particles accumulated in tumor tissue due to BBB disruption, the functionalized nanoparticles remained at the tumor site for significantly longer periods, up to 24 h.

The IL-13 receptor has also emerged as a promising target for brain tumor imaging. In a study by Li et al., IL-13-coated gadolinium metallofullerene nanoparticles demonstrated enhanced targeting of glioblastoma cells in the U251 GBM cell line [39]. In an orthotopic mouse model, these coated nanoparticles provided effective contrast delivery and selective tumor accumulation. Compared to Magnevist (Bayer Healthcare Pharmaceuticals Inc., Germany), a gadolinium-based MRI contrast agent, the IL-13-coated nanoparticles achieved sharper tumor border delineation, even at significantly lower concentrations. These findings underscore the potential of receptor-targeted nanoparticles in enhancing tumor resolution and improving the distinction between tumor and normal tissue.

Nanoparticle agents that integrate multiple imaging modalities are emerging as valuable tools in neurosurgical oncology, facilitating preoperative, intraoperative, and postoperative applications. These agents effectively address discrepancies between preoperative MRI and intraoperative findings due to brain shift while enhancing imaging sensitivity and specificity [40]. Kircher et al., pioneered the development of a triple-modality nanoparticle for MRI, photoacoustic imaging, and surface-enhanced Raman scattering (MPR) [41]. Intravenous accumulation of MPRs in orthotopic GBM mouse models demonstrated specific and prolonged retention in tumor sites for at least one week, allowing for utility in both preoperative and intraoperative settings. Each imaging modality contributed to precise tumor border delineation: MRI delineated margins preoperatively, while photoacoustic imaging provided high spatial resolution 3D imaging intraoperatively, achieving superior signal-to-noise ratios compared to fluorophores in deeper tissues. Additionally, Raman imaging offered highly specific, real-time imaging that facilitated fine margin resection and postoperative confirmation of clear margins. Although MPRs did not incorporate specific tumor-targeting mechanisms, their prolonged accumulation in tumors can be attributed to the enhanced permeability and retention (EPR) effect. Other multimodal nanoparticles have employed tumor-targeting agents, such as chlorotoxin, to establish specific targeting [29].

By leveraging the strengths of different modalities, these multimodal nanoparticle agents provide a comprehensive view of tumor margins, enhancing surgical precision and facilitating real-time decision-making during surgery. By minimizing the reliance on multiple contrast agents and fluorophores, these agents may increase efficiency in a surgical setting. We have summarized these current findings in Table 2 for our readers.

## 5. Emerging Uses of Theranostics in Stereotactic Radiosurgery

Radiation therapy, including radiotherapy and SRS, remains a cornerstone of brain tumor management. SRS is effective at treating a host of central nervous system malignancies, including primary and metastatic tumors, meningiomas, vestibular schwannomas, and trigeminal schwannomas [30,42,43,44]. However, tumor cells often develop resistance to radiation due to intrinsic and microenvironmental factors such as hypoxic regions, upregulated DNA damage response pathways, altered cell cycle dynamics, and protective signaling mechanisms [31]. Radiosensitizers have shown potential in overcoming these barriers by amplifying radiation-induced damage in tumor cells through both direct physical mechanisms and by targeting radioresistance pathways. Despite their promise, conventional radiosensitizers often lack specificity for tumor cells, limiting the radiation dose that can be safely administered without causing significant damage to surrounding healthy tissue. The development of radiosensitizers that selectively target tumor-specific receptors represents a promising strategy to enhance therapeutic efficacy while minimizing off-target toxicity.

Some nanoparticle radiosensitizer formulations utilize the EPR effect to facilitate targeted accumulation at tumor sites. 5-iodo-2-deoxyuridine (IUdR) is a conventionally used radiosensitizer effective in GBM; however, its short circulation time and limited ability to penetrate the BBB restrict its clinical application [32]. To address these limitations, Shirvalilou and colleagues encapsulated IUdR in magnetic graphene oxide nanoparticles coated with poly(lactic-co-glycolic acid) (PLGA) (IUdR/MNPs). In a rat C6 glioma model, IUdR/MNPs demonstrated enhanced BBB penetrability, specific accumulation, and prolonged retention at tumor sites, despite the absence of active targeting strategies. When combined with 8 Gy radiation, tumor growth was significantly inhibited, survival was markedly extended, and the anti-apoptotic response was significantly reduced, evidenced by a 6.2-fold increase in the Bax/Bcl-2 ratio compared to radiation alone. IUdR/MNPs achieved a dose enhancement factor of 2.26, indicating potent radiosensitization [32].

Other radiosensitizer nanoparticle formulations target tumor-specific receptors. The folate receptor has emerged as a promising target for radiosensitizers. Kefayat et al., designed folic acid and bovine serum albumin-decorated gold nanoclusters (FA-AuNCs) targeted toward folate receptors [45]. In the C6 glioma cell line, inductively coupled plasma optical emission spectrometry measurements revealed a 2.5-fold increase in FA-AuNC uptake in tumor cells compared to normal cells. Furthermore, in an intracranial rat model of GBM, significantly higher concentrations of FA-AuNCs were observed in brain tumors relative to the surrounding normal tissue. FA-AuNCs exhibited a dose enhancement factor of 1.6 Gy when irradiated with a single dose of 6 Gy, leading to increased overall survival compared with the control group, thereby underscoring their potential as effective targets for radiosensitizers.

A study by Séhédic et al., identified the chemokine receptor CXCR4 on glioma stem-like cells (GSCs) as a potent target for radiosensitizers [46]. GSCs play a critical role in glioma progression, driving tumor initiation and recurrence through self-renewal and differentiation capabilities, promoting therapeutic resistance due to enhanced DNA repair mechanisms and quiescent states, contributing to tumor heterogeneity, and facilitating invasion and angiogenesis [47]. In this study, the internal vectorized radionuclide rhenium-188 was encapsulated in a lipid nanocapsule and conjugated with the anti-CXCR4 antibody 12G5 (12G5-LNC188Re). In an orthotopic and xenogenic GBM mouse model, a single infusion of 12G5-LNC188Re, delivered via convection-enhanced delivery, resulted in significantly improved median survival and demonstrated locoregional effects on tumor development, including hypovascularization. This suggests that targeting CXCR4 on GSCs with specialized radionuclide delivery systems may offer a viable strategy for enhancing radiosensitivity and improving therapeutic outcomes in GBM treatment.

Low-density lipoprotein receptor-related protein 1 (LRP-1) is overexpressed on the BBB and glioma cells, providing a promising target for radiosensitizers. Zong et al., developed a lipid–polymer nanoparticle system, A2-P(MIs)25/TMZ, for the targeted delivery of TMZ to glioma cells [48]. This formulation incorporates Angiopep-2 (A2), which selectively targets LRP-1. Notably, under hypoxic conditions, the nitro groups of the hydrophobic P-(MIs)25 core are converted into hydrophilic amino groups (P(NH2s)25) through the transfer of six electrons, significantly enhancing DNA damage in tumor cells induced by ionizing radiation. In vitro studies using C6 glioma cells demonstrated selective accumulation of A2-P(MIs)25/TMZ and a potent radiosensitization effect, leading to increased cellular apoptosis. Furthermore, in a C6 xenograft mouse glioma model, this nanoparticle formulation exhibited specific accumulation at tumor sites, effectively inhibiting glioma growth and improving survival time without causing adverse effects. The combination of targeted radiosensitizers with established therapies such as TMZ exemplifies a promising theranostic approach that enhances treatment efficacy while minimizing systemic toxicity. This synergistic approach holds potential for overcoming the inherent resistance mechanisms which characterize brain malignancies. We have summarized this section for our readers in Table 3.

**Figure 4:**
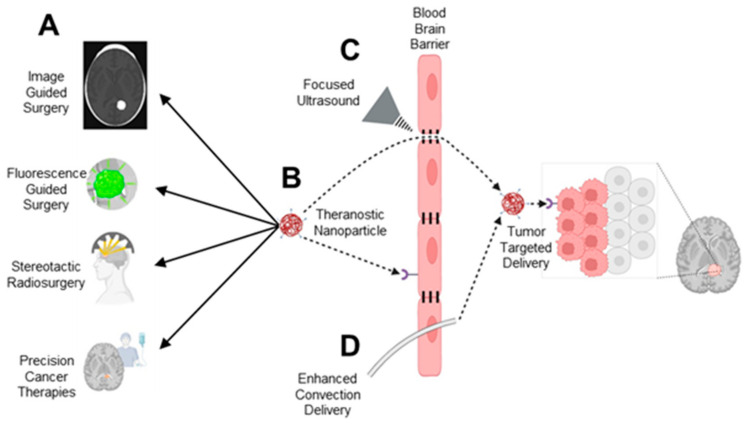
Leveraging theranostic nanotechnologies to compliment the treatment of brain tumors. (**A**) Current modalities used for treating brain tumors. (**B**) Theranostic nanoparticles can potentially augment these treatment modalities. Tools to enhance the delivery of nanoparticles across the blood brain barrier such as: (**C**) Focussed ultrasound, and (**D**) Enhanced convection delivery, can allow for tumor-targed delivery of novel combination therapies to increase tumor cell killing.

In summary, nanoparticle-based delivery systems (Figure 4B) can be broadly applied across multiple brain tumor treatment modalities (Figure 4A), potentially addressing these issues by enhancing targeting accuracy, improving tumor margin delineation, and broadening applicability across a variety of CNS tumors. An early-phase clinical trial using pegylated nanoliposomal irinotecan combined with metronomic TMZ was tested in recurrent GBM patients, though without specific tumor-targeting mechanisms, a further indication of the gradual coming of age of theranostic technology in the field of neuro-oncology [49]. The modular nature of theranostics also allows us to deliver multimodal therapies that can be both additive and/or synergistic in their tumoricidal effects when combined with other less invasive surgical treatments such as laser interstitial thermal therapy [50,51,52]. We can further leverage existing tools for BBB disruption such as focused ultrasound technology (Figure 4C) and convection-enhanced delivery (Figure 4D) to optimize therapeutic delivery.

**Table 3:** Targeted radiosensitizers to facilitate stereotactic radiosurgery.

Study	Population	Receptor Target	Intervention	Control	Key Results (Intervention vs. Control)
Shirvalilou et al. (2020) [46]	In vitro: C6 glioma cell line In vivo: C6 orthotopic glioma rat model	None (EPR effect)	Magnetic graphene oxide nanoparticles, coated with poly(lactic-co-glycolic acid), encapsulated with IUdR (IUdR/MNPs)	(1) IUdR (2) MPNs (3) no treatment	In vitro Significantly reduced IC-10 (*p* < 0.01) and IC-50 (*p* < 0.05) In vivo Specific accumulation and prolonged retention at tumor sites When combined with 8 Gy radiation Tumor growth significantly inhibited (101% vs. 97.27% vs. 94.03% growth inhibition rate, *p* < 0.001) Survival markedly extended (165 ± 22 vs. 165 ± 22 (1) vs. 165 ± 22 (2) vs. 20.5 ± 8 days (3), *p* < 0.001) Anti-apoptotic response significantly reduced (6.2-fold increase in Bax/Bcl-2 ratio compared to radiation alone) Potent radiosensitizer—dose enhancement factor of 2.26
Kefayat et al. (2019) [47]	In vitro: Rat C6 glioma cell line In vivo: C6 orthotopic GBM mouse model	FR	Folic acid and bovine serum albumin-decorated gold nanoclusters (FA-AuNCs)	Normal cells/tissue	In vitro Significantly greater uptake in tumor cells vs. normal cells (10.3 ± 2.2 vs. 4.7 ± 0.6 mean fluorescence intensity, *p* < 0.05) In vivo Significantly higher concentration in brain tumors compared to normal cells (8.1 μg/mg vs. 4.3 μg/mg, *p* < 0.05) Dose enhancement factor of 1.6 with single irradiation dose of 6 Gy
				Radiotherapy only	In vivo Significantly higher overall survival (25.0 ± 1.5 days vs. 18.3 ± 1.0 days, *p* < 0.001)
Séhédic et al. (2017) [48]	U87MG orthotopic and xenograft tumors in Scid mice	CXCR4	12G5-conjugated lipid nanocapsule encapsulated with rhenium-188	Blank lipid nanocapsules (LNCs), saline solution	Major improvement in median survival (74 days vs. 34 days for blank LNCs, 38.5 days for saline solution, *p* < 0.001) Regional hypovascularization, higher CD11b+ and CD68+ infiltrate
				Non-conjugated lipid nanocapsules encapsulated with rhenium-188	No significant difference in survival (74 days vs. 48 days)
Zong et al. (2019) [50]	In vitro: C6 glioma cell line In vivo: C6 orthotopic glioma mouse model	LRP-1	Angiopep-2-conjugated lipid–polymer nanoparticles encapsulated with temozolomide (A2-P(MIs)25/TMZ) or doxorubicin (A2-P(MIs)25/DOX)	Non-conjugated lipid-polymer nanoparticles encapsulated with doxorubicin (P(MIs)25/DOX)	In vitro Greater uptake of NPs in tumor cells Potent radiosensitizer—significantly increased γ-H2AX staining (a marker for double-stranded breaks) In vivo Significantly higher accumulation at tumor site (*p* < 0.01) Efficacy in accumulating in tumor hypoxic regions Strong inhibition of glioma growth
				(1) A2-P(MIs)25 + RT (2) A2-PLGA/TMZ + RT	In vivo Significantly higher inhibition of glioma growth (*p* < 0.01) Enhancement of apoptosis Significantly longer survival time (67 days vs. 44 days (1) and 48 days (2))

## 6. Current Limitations and Future Directions

While theranostic technologies hold considerable promise, several limitations must be acknowledged. Immunogenicity remains a significant concern, particularly with the use of viral vectors and bacteriophage-based systems [53]. Nanoparticle-based delivery systems can also elicit varying effects on the innate immune response, with the potential to induce both immune overactivation and immunosuppression [36]. Furthermore, the ultra-small size and large surface area of nanoparticles, while facilitating receptor interactions at the tumor site, also promotes organ accumulation and mediates toxicity. Nanoparticles have been associated with the accumulation of reactive oxygen species (ROS), mitochondrial damage, inflammation, cellular apoptosis, and DNA damage across a variety of organ systems including the respiratory, nervous, endocrine, and reproductive systems [37]. Continued research is necessary to fully understand the pharmacological properties and long-term effects of nanoparticle therapies in humans.

Moreover, the transition from benchtop to bedside presents significant challenges. While preclinical models have demonstrated success in overcoming the BBB and achieving targeted delivery, translating this into clinical practice remains complex. This complexity arises in part from the inability of preclinical models to fully replicate the heterogeneity of human tumors, particularly in terms of the tumor microenvironment and BBB characteristics [38]. Finally, the scalability and reproducibility of nanoparticle manufacturing processes also poses a substantial challenge, exacerbated by regulatory requirements and the high financial barriers associated with the development, testing, and production of theranostic technologies [39]. Nevertheless, the growing involvement of clinical scientists in nanotherapeutics research will inevitably accelerate the translation of these innovations into clinical settings to improve the treatment and survival outcomes for brain tumor patients.

## Figures and Tables

**Table 1:** Targeted fluorophores to facilitate fluorescence-guided neurosurgery.

Study	Population	Receptor Target	Intervention	Control	Key Results (Intervention vs. Control)
Patil et al. (2019) [17]	17 adults with gloma (9 high-grade, 8 low-grade)	MMP1/2	BLZ-100 (CTX-conjugated ICG)	None	No dose-limiting toxicity Retention in tumors > 24 h 12/17 tumors demonstrated positive fluorescence on ex vivo imaging
Warram et al. (2015) [29]	3 (2 = GBM, 1 = grade 2 diffuse astrocytoma)	EGFR	Cetuximab-IRDye800	None	Only the 2 patients with contrast enhancing tumors showed intraoperative fluorescence On paraffin-embedded tissue, fluorescence strongly correlated with histological evidence of tumor Fluorescence for tumor detection had a sensitivity of 73.0% for 50 mg dose, 98.2% for 100 mg dose; and a specificity of 66.3% for 50 mg dose, 69.8% for 100 mg dose
Elechala-war et al. (2019) [30]	In vitro: GL261 glioma cell line In vivo: GL261 orthotopic and subcutaneous xenograft tumors in C57BL/6 J mice	FR (Folate receptor)	Folic acid cationic lipid conjugated carbon nanospheres loaded with DOX (CSP-F8-DOX/CFD)	Non-conjugated carbon nanospheres loaded with DOX (CSP-DOX/CD)	In vitro: Significantly increased cellular uptake in tumor cells, with significant inhibition in the presence of folic acid (*p* < 0.05) In vivo: Significantly higher accumulation at all time points tested (*p* < 0.05) with highly specific localisation to tumor sites
Huang et al. (2012) [31]	Transgenic GBM mouse model (RCAS-PDGF-driven/tv-a GBM) U-87MG orthoptic xenograft GBM mouse model TS543 orthoptic xenograft GBM mouse model	Integrin receptors	IRDye 800CW-RGD	Non-fluorescent cyclic RGD peptide (cRGD)	Highly specific localisation to GBM tissue with overexpressed integrin receptors
				IRDye 800CW-RAD (non-specific)	Precise delineation of tumor tissue across all 3 mouse models (high tumor-to-normal brain ratio, *p* < 0.01, with maximal ratio at 48 h) Low background signal, high signal-to-background ratio Facilitated fluorescence-guided GBM resection
Li et al. (2018) [32]	U87MG orthotopic xenograft tumor in athymic mice 14 GBM patients	GRPR	68 Ga-IRDye800CW-BBN	None	In mouse model, clear visualization of tumor margins facilitating complete resection High correlation between preoperative PET uptake and intraoperative fluorescence signal Tumor fluorescence signals significantly higher than adjacent brain tissue (*p* < 0.0001) Fluorescence for tumor detection had a sensitivity of 93.9% and a specificity of 100%

**Table 2:** Targeted theranostic nanoparticles designed to facilitate image-guided neurosurgery.

Study	Population	Receptor Target	Intervention	Control	Key Results (Intervention vs. Control)
Sun et al. (2008) [33]	In vitro Rat 9L/lacZ glioma (9L) and human D283 medulloblastoma (D283) cell lines In vivo 9L flank xenograft tumor in athymic (nu/nu) mice	MMP1/2 (matrix metalloproteinase receptor 1/2)	CTX-conjugated iron oxide nanoparticles	Non-conjugated iron oxide nanoparticles	In vitro Higher contrast agent relaxivity, (r_2_ = 637.8 s^−1^mM^−1^ vs. 185.6 s^−1^mM^−1^) Higher internalization by 9L and D283 cells (approximately 3-fold) as indicated through T2 In vivo More thorough highlighting of tumors one day post injection Three days post injection, T2 remained at the decreased level vs. recovered to preinjection level
Na et al. (2007) [34]	Breast cancer brain metastatic tumor in mice	EGFR (epidermal growth factor receptors)	Herceptin-conjugated MnO nanoparticles	Non-conjugated MnO nanoparticles	More selective enhancement of tumor cells on T1 MRI with clear marginal detectability Longer accumulation at the tumor site, up to 24 h
Li et al. (2015) [37]	In vitro U-251 GBM cell line In vivo U-251 orthotopic xenograft tumor in athymic mice	IL-13 (interleukin 13 receptor)	IL-13-coated gadolinium metallofullerene nanoparticles	Scrambled IL-13 peptide analog-coated gadolinium metallofullerene nanoparticles	In vitro Specific internalization by tumor cells whilst no internalization of control Higher contrast agent relaxivity at all magnetic field strengths in comparison to commercial contrast agents Magnevist and Omniscan In vivo Specific targeting of brain tumor models, facilitating MR visualization at relatively low concentrations
Kircher et al. (2012) [39]	TS543 orthotopic primary human xenograft glioblastoma mouse model	None (EPR effect)	Triple-modality magnetic resonance imaging–photoacoustic imaging–surface-enhanced Raman scattering (SERS) nanoparticle (MPR)	None	Clear visualization of tumor with all 3 modalities Imaging modalities strongly correlated with each other and with immunochemistry findings, indicating accurate delineation of tumor margins Photoacoustic and Raman signals facilitated tumor resection
Veiseh et al. (2009) [40]	In vitro 9L rat gliosarcoma cell line In vivo Transgenic mouse model, ND2:SmoA1, closely resembling human medulloblastoma	MMP1/2	CTX- and NIR fluorophore (Cy5.5)-conjugated iron oxide nanoparticle (NPCP-CTX)	Non-conjugated iron oxide nanoparticle (NPCP)	In vitro A 6.1 ± 1.1-fold increase in tumor cell uptake (*p* < 0.0001) In vivo Specific accumulation at tumor site (significant increase in r_2_) with minimal accumulation in healthy brain tissue Regions highlighted on MR strongly correlate with those identified in histological tissue slices stained with haematoxylin and eosin Significant NIRF signal at tumor site at both 2 and 120 h post injection, which strongly correlates with MR imaging and histological analysis
